# The Condition of the Uterus Five Weeks after Delivery

**Published:** 1874-11

**Authors:** Wm. F. Jefks


### The Condition of the Uterus Five Weeks after Delivery.

By WM. F. JEFKS, M. D.

An opportunity was lately afforded me of examining the condition of the uterus five weeks after delivery, when the following questions suggested themselves for investigation in the study of the specimen.

1st. From what tissue does the newly developed muscular fibre take its origin?

2d. Is the process of fatty degeneration completed at this date?

3d. What is the condition of the mucous membrane?

4th. How far have the bloodvessels taken part in the changes which the uterus has undergone?

After delivery, as we know, the gigantic muscular fibre cells undergo fatty degeneration and are absorbed, and in their place we find myriads of newly created cells, having all the characteristics of unstriped muscular fibres. From what source does this renewal of tissue take place? This investigation is exceedingly difficult, because there are few questions in histology more delicate than the diagnosis of smooth muscular fibre from the spindle-shaped connective tissue cell. The distinguishing mark usually relied on is the shape of the nucleus, which, in the first, is an elongated oval, with rounded extremities, while that of the connective tissue cell is drawn out into sharp prolongations at both poles. While this is usually true, the exceptions are numerous, and Prof. Arnold, of Heidelberg, who has won especial distinction by his investigations regarding the unstriped musbular fibre, confesses that while the nucleus is generally rounded, still it may be pointed at one or both poles, so that practically this fine theoretical distinction can hardly avail us in studying an individual cell. The fact, however, that muscular tissue is stained yellow by piric acid, while the connective tissue is not affected by this agent, assisted me in the study of this specimen; and I have been led to coincide with the opinion now held by some of the best histologists, that the reproduction is effected through the medium of the connective tissue cell in a way with which we are familiar from the study of the process of repair in other organs, viz: by their proliferation and division, forming thereby indifferent or embryonic cells, which subsequently develop into the unstriped muscular fibre. I have been led to this conclusion from the fact that I can find no evidence of the division of the nucleus of the fatty degenerated muscular cell, while it is contrary to the laws of reproduction to suppose that a cell, itself undergoing death by molecular fatty degeneaation, should manifest the highest attribute of life, viz: the production of a new cell endowed with all the marvelous attributes of vitality. In studying the process of fatty degeneration in other organs, we find that the nucleus, although retaining its function and form longer than the protoplasm, does itself in turn succumb, and finally is converted into fatty molecules, which may subsequently be absorbed. The parenchyma of the uterus submitted to me for examination was, however, infiltrated with small round young cells, such as we meet with in the repair of every tissue after injury, to which the name of embryonic or indifferent cells have been given, because they are impressed with the type of the tissue in which they are generated, and are capable, like the cells which we find in the germinal mass or vitelline sphere in the embryo, of development into muscular nervous or osseous tissue. On examining the external muscular layers of the organ, the remains or the old hypertrophied muscular fibre were evident, the individual cells, however, were much diminished in size and filled with fatty granules; but nowhere else in the uterus was there a trace to be found of the former muscular structure; hence the inference is rendered probable that the process of rejuvenation proceeds from within outwards, and approaches completion at or near the fifth week after parturition, and this opinion coincides nearly with that of Heschel, who states that the fatty degeneration and absorption of the old muscular structure is not completed before, nor does it continue after the eighth week. Priestly, in his treatise on the "Gravid Uterus," writes that the colossal muscular fibres are not found after the third week, the middle coat now consisting of embryonic cells.

The view of Cruveilhier, that after delivery the entire mucous membrane of the uterus is thrown off, so that the inner surface of the organ resembles a stump after amputation, is now generally abandoned, a delicate layer of decidua cells always remaining, from which the reproduction of the new mucous membrane takes place, the process commencing even during the later months of pregnancy. In this specimen the newly formed mucous membrane was everywhere apparent, except over the placental site, which still projected somewhat above the general surface, the bloodvessels filled with the contracted physiological thrombi. The glandular structure was also reproduced in this new lining membrane. This hypertrophy and subsequent degeneration of muscular tissue was not confined alone to the parenchyma of the organ, for the muscular coat of the arteries showed also, here and there, cells in a state of fatty degeneration, and newly developed embryonic cells.

My attention has been especially directed to this microscopic examination, from the fact that at present the dietetic management of the puerperal state is a question which is being earnestly discussed. I cannot but believe that, during the time when this active process of involution is taking place, not only in the uterus, but also in the walls of the vagina, and the ligaments which aid in supporting the womb, while the organ itself is greatly increased in weight with diminished tonicity, a rest in bed for ten days or two weeks, and subsequently a careful return to any active exercise, is plainly indicated. In this opinion I am the more convinced from the fact that in a dispensary practice, where the patients are drawn from the poorer class in society, unable or unwilling to submit to restraint after confinement, by far the commonest form of uterine disease is subinvolution of the uterus after delivery or abortion, with its attending ills of displacements, and chronic catarrhal conditions of the mucous membrane.

These are essentially chronic conditions, slowly developed, depending for their existence on structural changes in the tissues, or rather on the *arrest* of certain changes which, phisiologically, should take place, which are not, however, normally completed until the expiration of a month or more after delivery. Therefore, in estimating the value of any mode of treatment of the puerperal woman, her condition, six months or a year after her confinement, must be the criterion, and not her general health a month after delivery. Even if this condition of subinvolution exists, the physical signs and symptoms are manifested only when the enlarged and indolent organ, engorged in consequence of a sluggish circulation—which is in part due to the implication of the muscular tissues of the bloodvessels in this arrested repair—sinks deeper into the cavity of the pelvis. The uterus usually becomes retroverted as it descends, inasmuch as it does not receive the proper support from the relaxed ligaments, vaginal walls, and perineal body, while the mucous membrane, owing to the passive congestion of the bloodvessel system, passes into a state of chronic catarrh, and the accompanying disturbances, both local and sympathetic, from this enlargement, structural change. and displacement of the organ, slowly but surely develop themselves at a later period.—*Obst. Journal, Gt. Britain and Ireland. Am. Supplement.*

